# Deterministic, random, or in between? Inferring the randomness level of wildlife movements

**DOI:** 10.1186/s40462-021-00273-7

**Published:** 2021-06-29

**Authors:** Teresa Goicolea, Aitor Gastón, Pablo Cisneros-Araujo, Juan Ignacio García-Viñas, M. Cruz Mateo-Sánchez

**Affiliations:** ETSI Montes, Forestal y del Medio Natural, Universidad Politécnica de Madrid, Ciudad Universitaria s/n, 28040 Madrid, Spain

**Keywords:** Connectivity validation, Ecological connectivity, Ecological corridors, Iberian lynx, *Lynx pardinus*, Movement ecology, Point selection functions, Randomized shortest path, Corridors

## Abstract

**Background:**

When assessing connectivity, it is crucial to rely on accurate modeling frameworks that consider species movement preferences and patterns. One important aspect is the level of randomness or unpredictability in the route selection. In this respect, traditional approaches (based on least-cost path or circuit theory) consider species movements unrealistically as totally deterministic or as totally random. A recent approach (randomized shortest path) advocates for choosing intermediate levels of randomness through a single parameter. This parameter may be optimized by validating connectivity surfaces developed from different levels of randomness against observed movement data. However, connectivity models are seldom validated, and it is still unclear how to approach this task. To address this knowledge gap, this paper aims at comparing different validation methods to infer the optimal randomness level in connectivity studies. Additionally, we aimed to disentangle the practical consequences of applying traditional connectivity approaches versus using an optimized level of movement randomness when delineating corridors.

**Methods:**

These objectives were accomplished through the study case of the Iberian lynx, an endangered species whose maintenance and recovery depend on the current connectivity among its population nuclei. We firstly determined a conductance surface based on point selection functions accounting for the behavioral state (territorial or exploratory) of individuals. Secondly, we identified the level of randomness that better fits lynxes’ movements with independent GPS locations and different validation techniques. Lastly, we delineated corridors between lynx population nuclei through a) the randomized shortest path approach and the extreme and optimal levels of randomness of each validation method, and b) the traditional connectivity approaches.

**Results:**

According to all used validation methodologies, models with intermediate levels of randomness outperformed those with extreme randomness levels representing totally deterministic or random movements. We found differences in the optimal randomness level among validation methods but similar results in the delineation of corridors. Our results also revealed that models with extreme randomness levels (deterministic and random walk) of the randomized path approach provided equivalent corridor networks to those from traditional approaches. Moreover, these corridor networks calculated with traditional approaches showed notable differences in patterns from the corridor network calculated with an optimized randomness level.

**Conclusions:**

Here we presented a connectivity model with a solid biological basis that calibrates the level of movement randomness and is supported by comprehensive validation methods. It is thus a step forward in the search and evaluation of connectivity approaches that lead to improved, efficient, and successful management actions.

**Supplementary Information:**

The online version contains supplementary material available at 10.1186/s40462-021-00273-7.

## Background

Biodiversity planning is increasingly focused on enhancing ecological connectivity, i.e., the degree to which the landscape facilitates or impedes movement among habitat patches [1], as it has been shown to counteract fragmentation and soften its detrimental effects [2–4]. Connectivity analyses contribute to the identification of priority areas and measures to preserve and enhance biodiversity [5, 6]. However, there is still no consensus about which is the best methodology to follow when studying connectivity, as it may vary with the species and objective of the study [7, 8].

Connectivity modeling is generally based on a resistance surface or its inverse, a conductance surface [9] that reflects the influence of landscape factors and habitat selection in species movements. One of the most advocated ways to obtain them is through Resource Selection Functions trained with telemetry data [10]. Resource Selection Functions encompass several models that predict the likelihood of selecting each landscape cell by comparing the habitat attributes of the used cells by the studied individuals with the attributes of the unused but available cells. Among Resource Selection Functions, Point Selection Functions (PSF) are one of the most common ways to estimate resistance surfaces [9, 11–15]. However, habitat selection for animal movement does not only depend on the focal species and the habitat around the location of individuals [16], as it can also be influenced by the demographic factors [17] and the behavioral state of individuals [14, 18]. Acknowledging these additional conditions in PSF contributes to a more thorough understanding of the relationship between species and environmental factors [19] and promotes the attainment of more realistic resistance or conductance surfaces as input for connectivity analyses.

Many methods based on resistance models are available to characterize the possibilities for species movement [20]. Among the existing techniques, least-cost path (LCP) [21, 22] is one of the most used approaches to evaluate the preferential movement pathways. It identifies the pathway with the minimum accumulated cost of movement or effective distance between the source and destination points. Even though this approach is broadly accepted and used, it is also deterministic, as it considers that animals have a perfect knowledge of the landscape and can determine and follow the optimal path [23] in terms of effective distance. In order to relax this optimum single path assumption, many connectivity analyses started to focus on an approach based on the circuit theory [24]. This framework considers all possible pathways between each pair of nodes in terms of a weighted random walk, assuming only knowledge of the immediate surrounding landscape (i.e., only knowledge of the underlying resistance/conductance values of the immediate neighbor cells).

LCP and circuit theory methods have been widely adopted [3, 6, 20, 25–27], although they might be based on unrealistic assumptions as animals generally follow an intermediate strategy between totally deterministic and random movements [28]. To surmount this issue, a novel approach has been proposed, the Randomized Shortest Path (RSP) [29–32]. This method allows considering deterministic and random movements as well as all intermediate states between them, by calibrating the level of randomness (parameter *θ*). A weighted random walk is assumed (comparable to circuit theory approaches) when *θ* is set to 0. However, increasing *θ* values imply more deterministic movements (comparable to LCP). This and other model parameters should be adapted to each study case and species movement traits to assess functional connectivity [16, 33, 34]. Traditional connectivity models only take into account species’ habitat preferences and dispersal abilities (i.e., dispersal distances) to model functional connectivity, while RSP models additionally consider the specific level of movement randomness.

How to identify the level of randomness that best represents each species movements is still fairly unknown. Most studies performing RSP [29, 35–38] addressed this issue by validating connectivity models deriving from a range of different *θ* values with an independent set of movement data. The model with the best validation results represented to a larger extent the actual movements of the organisms and established the optimal *θ*. However, most connectivity studies do not validate their results [3, 14], and so far, scientists have not concluded which is the best validation approach. There are many validation techniques [8, 11, 14, 39–44], and using several methods might be desirable [7, 13] as they can be complementary and inform about different aspects. To our knowledge, studies applying RSP identified the optimal *θ* through a similar validation technique [29, 35, 36] and so far none have compared the practical results (i.e., identification of conservation corridors) from different validation methods. However, applying other validation methods to optimize *θ* might produce different and complementary results. Therefore, further research in this direction is still necessary due to the far-reaching consequences for conservation and management.

The main aims of this work were a) to conduct a comparative research on different validation methods and assumptions when inferring the randomness level in animals’ movements; and b) comparing the performance of connectivity modeling approaches (particularly RSP, LCP, and circuit theory). We also produced an improved landscape connectivity model accounting for the species movement characteristics: i) behavioral state (territorial or exploratory states); ii) preference of habitat for movement (with PSF); and iii) inferred level of randomness. Lastly, we defined the species network of corridors based on the most reliable information. We used the Iberian lynx (*Lynx pardinus*) as the focal species due to the availability of extensive and high-quality monitored data (GPS records) and the importance of the connectivity of this endangered species for its long-term conservation. Together, these analyses provided sound information to support practical guidance on Iberian lynx conservation and fill the gaps in the general knowledge around connectivity modeling.

## Materials and methodology

### Study data and species

**Fig. 1:**
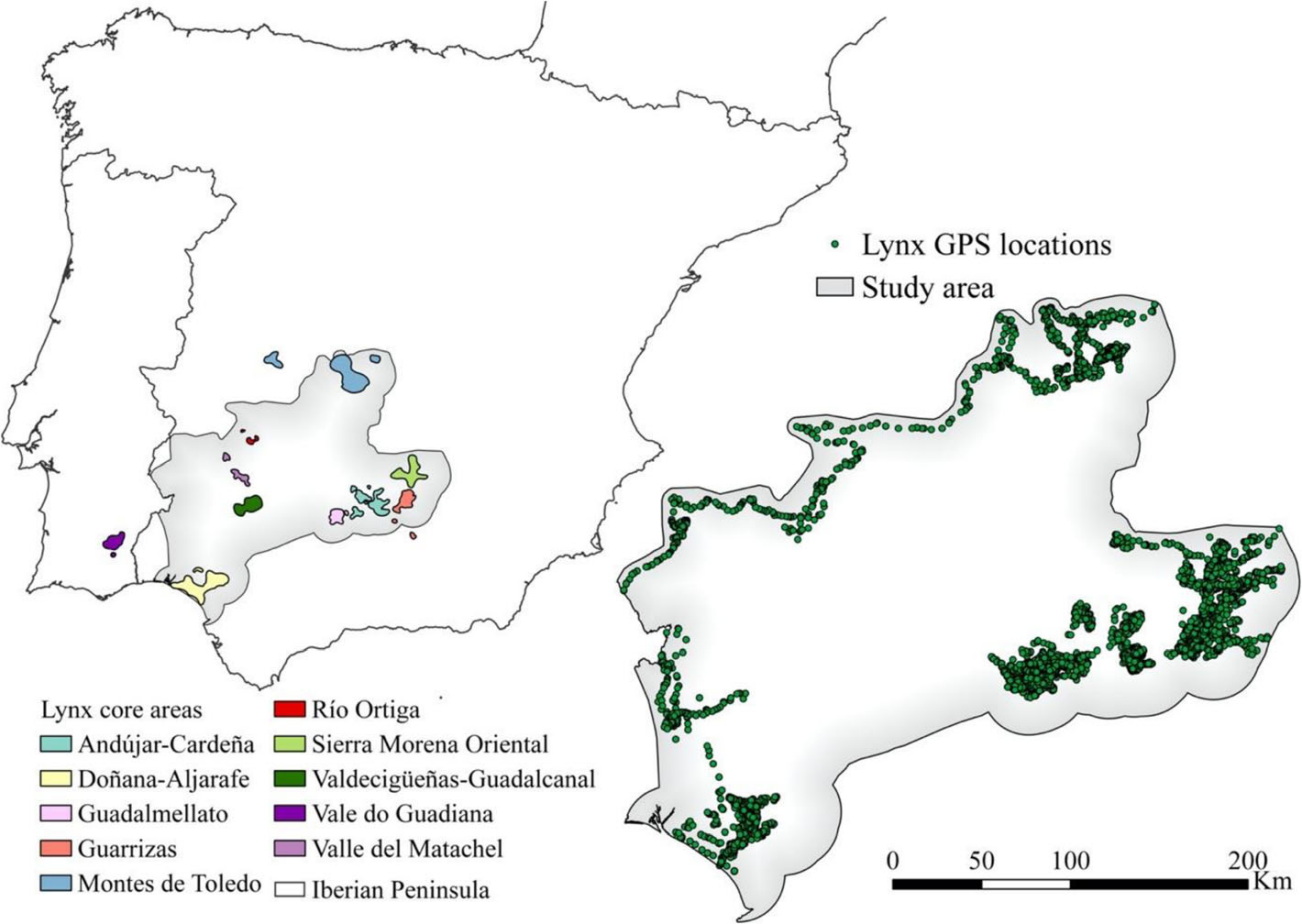
Study area location in the Iberian Peninsula, known lynx nuclei, and GPS locations

Iberian lynx is an endangered species from the Iberian Peninsula [45] whose population was at its minimum in 2002 [46]. Habitat loss and fragmentation, landscape change, hunting, and lack of prey were the main threats that led to the steep decline of the Iberian lynx populations [47]. However, this decreasing trend was reversed thanks to intensive monitoring, conservation, and breeding efforts [46], which have succeeded in increasing lynx numbers in their historical and new population nuclei. Nonetheless, the connectivity between those nuclei is critical to ensure long-term maintenance and recovery of their still fragile populations (Fig. 1).

The study was carried out in the southeast of Spain (Fig. 1) and occupies an area of 67,673 km^2^. It covers most of the known Iberian lynx distribution in Spain. The species is distributed in the study area around 20 nuclei (Fig. 1). There are only two more known lynx nuclei in Portugal and Spain outside the study area.

**Fig. 2:**
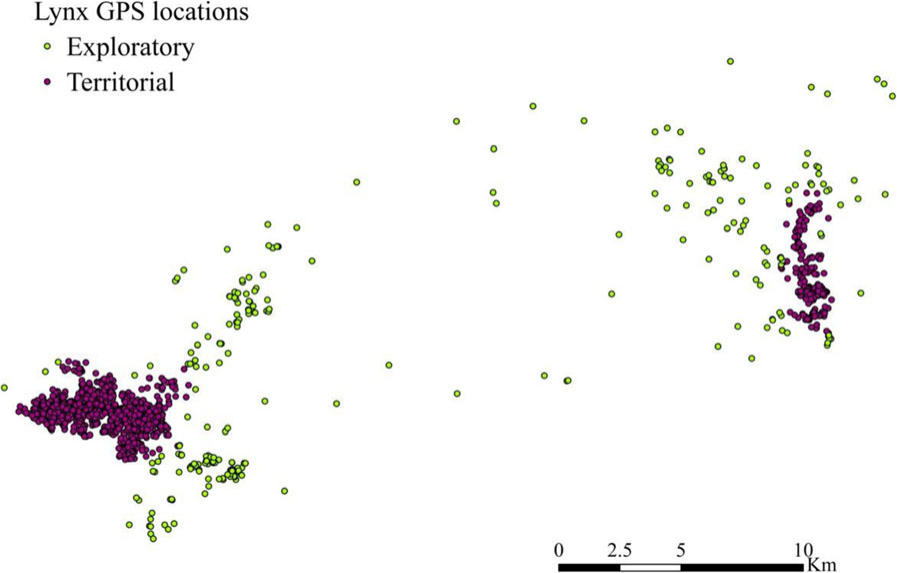
Example of the classification of the GPS locations into territorial (inside home ranges) and exploratory (outside home ranges) of one lynx

**Fig. 3:**
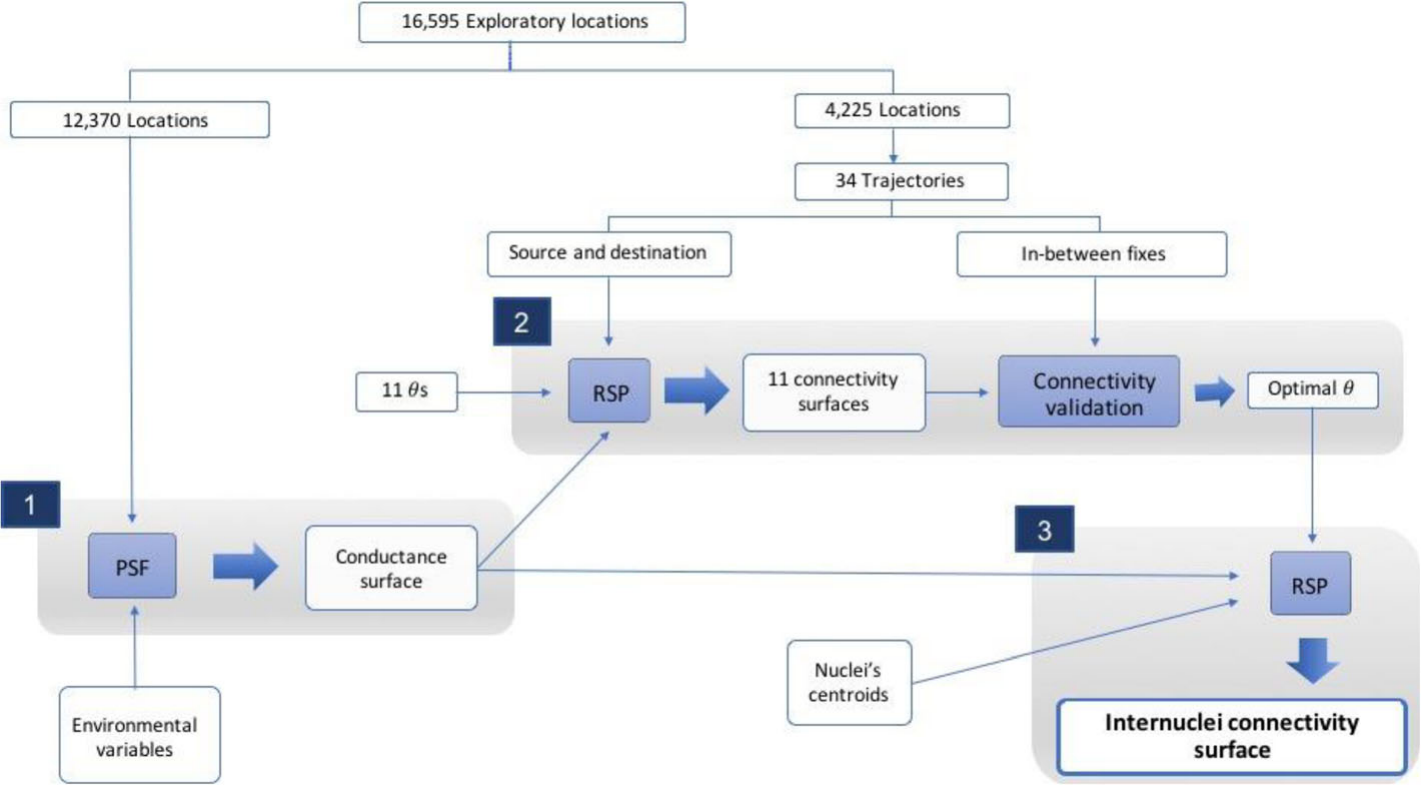
Methodological workflow scheme. Thin arrows represent inputs, whereas thick arrows outputs. We followed three steps: 1. Habitat selection modeling through PSF; 2. RSP calibration, creating 11 RSP connectivity surfaces and validating them to obtain the optimal *θ*. We used four different validation methods and obtained different optimal *θs*; 3. RSP between population nuclei. We lastly delineated corridors from the resulting internuclei connectivity surface of step 3. We compared the internuclei connectivity surfaces and corridors obtained through (a) RSP and the optimal *θ* obtained from every validation methodology considered, (b) RSP and the two extreme *θ* s (0 and 0.01), and (c) the two traditional connectivity approaches (least-cost path and circuit theory)

For the forthcoming analysis, we used 64,242 lynx locations obtained from 2008 to 2015 and provided by the project Life+IBERLINCE [48]. They were obtained from 67 individuals’ GPS collars every 4 h. Habitat selection by the Iberian lynx is known to depend on the behavioral state of the individual [49, 50], therefore lynx locations were classified into a) territorial, regular use of habitat within home ranges; and b) exploratory, corresponding to movements outside territorial areas [19, 51] (Fig. 2). To separate territorial and exploratory locations, we used local convex hulls of adaptive radius (a-LoCoH) [52–54] for each lynx independently. This method delineates the home ranges as the union of the minimum convex polygons of each location (root point) and its nearest neighboring points using a non-parametric local convex-hull [55]. The nearest neighbor points are the GPS locations within an adaptive sphere of influence around the root point with a radius dependent on the number of neighboring points and their distance to the root point [52]. We computed lynxes’ home ranges with the *LoCoH.a* function of the *adehabitatHR* R package. The objective of this paper was to assess the connectivity between lynxes’ nuclei, therefore we only worked with the exploratory data, consisting of 16,595 GPS locations (25.83% of the lynx locations), from the 67 individuals. Finally, these data were classified into two independent datasets (see Fig. 3) i) one used in the *Habitat selection during dispersal* section, to train and internally test model predictive performance (12,370 GPS locations from 57 lynxes, 74.5% of the exploratory data); and ii) another dataset used in the *RSP calibration* section, to evaluate the connectivity models (4225 GPS locations from the remaining ten lynxes, 25.5% of the exploratory data). As the goal was to determine the randomness level of dispersal movements, the validation dataset consisted of the 10 lynxes with the longest inter-nuclei movements.

### Habitat selection during dispersal

Habitat selection during exploratory movements was estimated through PSF (step 1 in Fig. 3) [8, 11] following the methodology of 10,13,17, and using the subset of exploratory lynx locations intended for modeling habitat selection. Seventeen explanatory variables (Table 1) were used as predictors of lynxes’ movements. Predictors were derived from the combination of a high-resolution land cover map (LCM, developed in collaboration with Agresta Soc. Coop. using Sentinel 2 satellite imagery); with vegetation structure features derived from LiDAR [56]; with the slope from a digital elevation model (DEM); with the Dominant Leaf Type from Copernicus Land Cover Services (CLCS); and with roads from OpenStreetMap data. The resulting raster layer of each explanatory variable had a spatial resolution of 25 m.

**Table 1:** Explanatory variables included in the habitat preference model derived from the combination of a land cover map (LCM), LiDAR, a digital elevation model (DEM), Copernicus Land Cover Services (CLCS), and OpenStreetMap data. Coniferous trees and Broadleaved trees covers were split into four variables according to the percentage of tree canopy cover (TCC) to differentiate forest closures. The Unsuitable areas variable is composed of three land cover classes (urban areas, water bodies, and bare soil) with very few lynx GPS locations

Variables		Land cover class (regional LCM)	TCC (LiDAR)	Shrub canopy cover (LiDAR)	Slope (DEM)	Dominant Leaf type (CLCS)	Dataset
Unsuitable areas		Urban areas, water bodies, and bare soil	–	–		–	Regional Land Cover Map
Intensive crops		Irrigated herbaceous crops	–	–		–	
Extensive crops		Dry herbaceous crops	–	–		–	
Agroforestry		Agroforestry areas	> 25%	> 25%		–	Regional Land Cover Map, LiDAR
Lowland olive groves		Permanent woody crops	–	–	< 20%	–	Regional Land Cover Map, DEM
Mountain olive groves			–	–	> 20%	–	
Grasslands and Pastures		Forest and Pastures	< 5%	< 5%		–	Regional Land Cover Map, LiDAR
Shrublands			< 5%	> 5%		–	
Coniferous trees	Low TCC		5–25%	–		Coniferous	Regional Land Cover Map, LiDAR, CLCS
	Low-intermediate TCC		26–50%	–			
	Intermediate-high TCC		51–75%	–			
	High TCC		> 75%	–			
Broadleaved trees	Low TCC		5–25%	–		Broadleaved	
	Low-intermediate TCC		26–50%	–			
	Intermediate-high TCC		51–75%	–			
	High TCC		> 75%	–			
High-traffic roads		–	–	–		–	Open Street Map
Low-traffic roads		–	–	–		–	

We then compared the used (selected by the lynxes) and the available (reachable but presumably not selected) habitat. Habitat was characterized as the proportion of each land cover type covering the used and available areas around each GPS location: The used habitat corresponded to the proportion of each cover type in each lynx location and its eight neighboring pixels (to deal with GPS accuracy); The available habitat was estimated as the distance-weighted average of each cover type in a reachable circumference around the GPS location. The radius of this circumference (5.72 km) was selected from the 99th percentile of the straight-line distances between each pair of consecutive points. This available radius represented the maximum reachable distance in a 4-h step, omitting outliers. Weights were obtained from a generalized Pareto distribution [57] fitted to the empirical distribution of 4-h displacement distances [12], i.e., pixels closer to the GPS point had a larger weight than distant pixels. Using the used-available matched data, we fitted a conditional logistic regression model [58] with *glm* R function to estimate the probability of habitat selection with grasslands as the reference land cover class. To test the model performance, we conducted i) 10-fold cross-validation [59], and ii) spatial cross-validation [60]. The predictive performance of the model was assessed using the area under the receiver operating characteristic curve (AUC).

Model predictions were calculated for the entire study area, generating a raster surface with the suitability of each pixel for being used by exploratory lynxes. This surface was aggregated to a spatial resolution of 125 m to ease the forthcoming connectivity analysis. The resulting surface was used as a conductance surface, quantifying the matrix permeability, i.e., lynxes’ willingness to use each landscape unit for moving depending on the land cover [9].

### RSP calibration

We calibrated the randomness parameter to obtain a connectivity model with the adjusted level of randomness (step 2 in Fig. 3). For this purpose, we used the second dataset of exploratory locations that comprises the locations of ten lynxes that undertook long inter-nuclei movements. These locations were split into trajectories whenever they crossed a population nucleus, changed to territorial behavior, or turned abruptly (almost 360°) their general direction. A number of 34 trajectories were generated, with lengths ranging from 15 to 431 km (mean of 64 km and median of 41 km), and time lapses from 1 to 83 days (mean of 11 days and median of 4 days). RSP calibration consisted of two steps: a) Running a range of RSP models with different *θ* values; and b) *θ* optimization. The former step only required the source and destination points of the lynx trajectories, whereas the second step used all intermediate fixes in between the source and destination.

a) Range of RSP models

We firstly run several RSP models with different values of *θ* to reflect the whole range of movement types; from random (equivalent to circuit theory approach and *θ* = 0) to deterministic movements (similar to LCP approach and *θ* →  ∞ ). However, a total deterministic movement is very unlikely and thus *θ* < ∞ was used to represent the deterministic extreme. Additionally, the upper limit of *θ* depends on the size of the graph and the values of the conductance surface [29]. Given this study case, the maximum *θ* was set to 0.01, which represents a smoothed LCP. Eleven RSP models were run, each one with a different value of *θ*: 0, 5·10^− 7^, 1·10^− 6^, 5·10^− 6^, 1·10^− 5^, 5·10^− 5^, 1·10^− 4^, 5·10^− 4^, 0.001, 0.005, and 0.01, with increasing values corresponding to more deterministic movements. We performed RSP analyses with the *passage* function in *gdistance* R package [61]. Each trajectory was individually processed running RSP between its source and destination points, and the results were subsequently summed to obtain an overall current surface for all trajectories. This surface estimated the probability that an exploratory lynx traversed each cell of the study area given the source and destination points, the landscape conductance, and the level of randomness.

b) *θ* optimization

Afterward, we assessed the level of randomness that optimized the agreement between each derived RSP connectivity surface and the observed lynx movements. To do so, we used the in-between fixes of the 34 lynx trajectories and four different validation methods: (1) *Brownian bridge*; (2) *Representation in corridors*; (3) *Logistic regression*; and (4) *Ranking*. Although other validation methodologies have been used and adopted in previous connectivity studies [8, 14, 39, 41, 42, 44], here we only selected the most usually adopted methods that fitted our input information. Every validation methodology was conducted with the R software.

The first method (*Brownian bridge*) is the approach followed in most of the previous papers running RSP analysis [29, 35]. Brownian bridge models [62] were used to estimate the probability of using each cell in the movement between all consecutive GPS points assuming total randomization. We used the *BBMM* R package to calculate Brownian bridge surfaces. The resulting Brownian bridge surface was constrained to all the monitored in-between fixes, and therefore, we considered it represented the observed movement. The RSP surface that minimizes the mean square error (MSE) with the Brownian bridge surface would correspond to the optimum *θ*.

The other three *θ* optimization methodologies were calculated at the point level. Each GPS location was considered independently of the lynx or trajectory it belonged to, and only the point and its surroundings were examined. Used points were the cells where GPS fixes fell. For each used point, we selected a set of available points that could be chosen by individuals instead of the observed ones. These available points were the pixels inside a circumference centered in the GPS location and a radius of 6.68 km. This radius corresponded to the maximum distance traversed in 4 h by the ten lynxes (99th percentile of the straight-line distances between each pair of consecutive points). Available pixels were weighted by their distance to the GPS point according to a generalized Pareto distribution [57]. We only used the available points for the *Logistic regression* and *Ranking* methods.

The second considered validation methodology (*Representation in corridors*) quantifies how well corridors capture the real movement of the species [7, 13, 14, 40]. Corridors were derived from each connectivity surface as the areas with the highest likelihood of being used in the movement of the species. Corridors were delineated as percentiles 99, 95, 90, and 85 of the connectivity surface values. To measure the performance of corridors, we measured the percentage of used GPS points falling within the corridors for each connectivity surface (from each *θ*). We then selected the connectivity surface (and its associated *θ*) with the greatest percentage of observed points falling within its corridors.

The third method (*Logistic regression*) was based on PSF. We fitted a conditional logistic regression with *glm* R function to model point selection probability depending on the flux current for each connectivity surface (each *θ*). We hypothesized that pixels with a higher flux current would be more used than those pixels with low current values. Therefore, the used cells should have higher mean connectivity values than the available points. We then selected the *θ* with the best model using Akaike’s information criterion (AIC).

Lastly, we used the *ranking* method. It also assumes that the most suitable connectivity surface corresponds to higher differences between the flux current values at the used and available points. However, this method additionally considers the percentile position of the used cells compared to the available ones, instead of comparing mean values. This approach has been similarly used to validate connectivity models [7, 43]. We calculated the rank position of the used point in relation to the available ones for each connectivity surface. The *θ* whose associated used points were in a better ranking position was chosen as the optimal level of randomness.

### Corridors between population nuclei

Once identified the optimal *θ* values, we defined corridors between the population nuclei centroids (step 3 in Fig. 3). For this purpose, firstly we calculated the flux density between all possible combinations of starting and ending nuclei through (a) RSP and the optimal *θ* obtained from each validation methodology, (b) RSP and the two extreme *θ*s (0 and 0.01), and (c) the two traditional approaches (LCP and circuit theory). RSP flux densities were calculated with the *passage* function of *gdistance* R package [61]. The LCP approach was conducted with Unicor software [63], which calculated the cumulative density of LCP between all nuclei buffered by a kernel density following a Gaussian bell curve. We used Circuitscape software [64] for the circuit theory approach to predict the net flux intensity [24]. RSP models were based on the conductance surface, while LCP and circuit theory approaches used a resistance surface (reciprocal of the conductance). Secondly, we delineated the corridor network for all methodologies and *θ*s as the 10% of pixels (percentile 90) of the connectivity surfaces with higher predicted flux density excluding the nucleus areas. We compared the resulting corridors to those delineated with the optimal *θ* from the *Representation in Corridors* validation method (optimized corridors) using the percentage of shared corridor area. We assumed that these optimized corridors captured better the inter-nuclei movements than those calculated with the optimal *θ*s from other validation methods.

## Results

The habitat selection model performed well discriminating used from available habitat for exploratory movements (estimated AUC values of 0.89 and 0.87 when performing 10-fold and spatial cross-validation, respectively). Table 2 shows the regression coefficients for selecting each land cover class. Shrublands, open woodlands, and mountain olive groves were the preferred land cover classes during movement. On the other hand, urban areas, herbaceous croplands, and roads were mostly avoided by exploratory lynxes. The conductance map derived from the habitat selection model is shown in supplementary material, Fig. S1.

**Table 2:** Estimated the variable coefficients with standard errors and the proportion of the study area covered by each variable. Grasslands and pastures is the reference land cover class and thus it does not have an estimated coefficient nor standard error

Variables	Canopy cover	Estimate	Standard error	Proportion of area
Unsuitable areas	–	−6.13	0.42	3.85%
Intensive crops	–	−2.70	0.39	5.42%
Extensive crops	–	−3.93	0.31	13.48%
Lowland olive groves	–	1.21	0.19	14.80%
Mountain olive groves	–	2.35	0.19	4.16%
Grasslands and Pastures	–	–	–	13.31%
Shrublands	–	2.98	0.28	5.00%
Coniferous trees	< 5%	2.64	0.35	1.71%
	5–25%	1.60	0.32	1.79%
	26–50%	0.56	0.31	1.44%
	51–75%	−0.10	0.42	0.84%
Broadleaved trees	< 5%	1.89	0.21	16.50%
	5–25%	1.97	0.21	10.85%
	26–50%	1.74	0.28	4.01%
	51–75%	0.20	0.47	1.09%
High-traffic roads	–	−29.68	2.79	0.49%
Low-traffic roads	–	−48.90	2.55	1.27%
**AUC normal cross-validation**		**0.89**		
**AUC spatial cross-validation**		**0.87**		

**Fig. 4:**
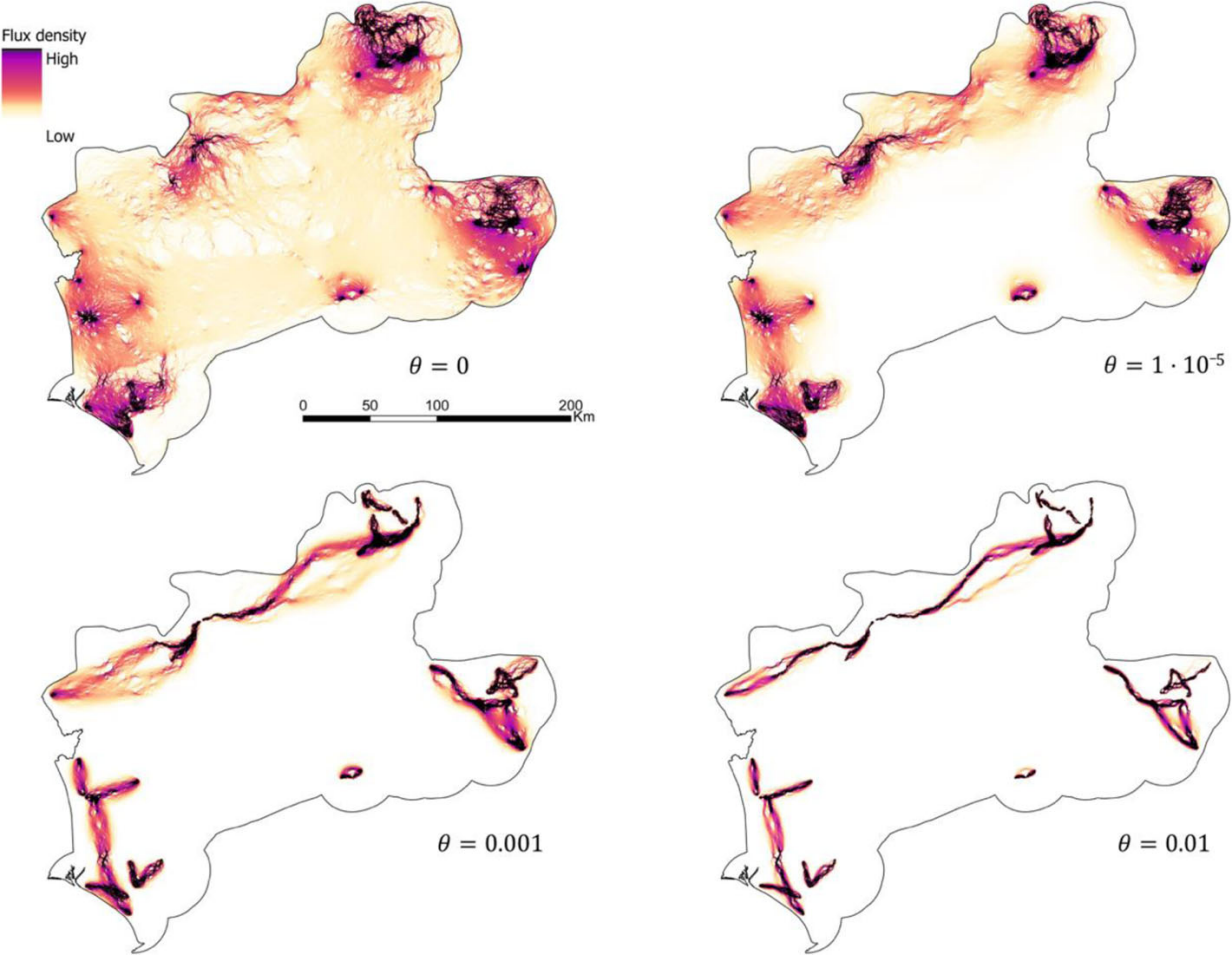
Connectivity surfaces calculated with four different *θ*s: 0 (random), 1·10^− 5^, 0.001, and 0.01 (deterministic). The values of all connectivity surfaces ranged from 0 to 2

We developed eleven RSP models with different *θ* values ranging from 0 (random movements) to 0.01 (deterministic movements). The resulting maps calculated with the extreme and two intermediate *θ*s are shown in Fig. 4, whereas the rest of the maps can be found in the supplementary material (Fig. S2). The values of all the resulting connectivity surfaces ranged from 0 to 2.

The optimal *θ* that maximized the agreement between observed and predicted movements fluctuated with the methodology followed (Table 3). In every considered case, the optimal *θ* was an intermediate value between the lower and upper limits. All methodologies presented better results for the lower limit of *θ* (0) than for the upper limit (0.01). Moreover, the optimal *θ*s tended to be very low (random) except for the Brownian bridge methodology. Details on the results of each optimization method can be found in supplementary material, Tables S3 to S6.

**Table 3:** Optimal theta according to each optimization method

Optimization method		Best *θ*
1. Brownian Bridge		1·10^− 4^
2. Representation in corridors	Percentile 85	5·10^−6^
	Percentile 90	5·10^−6^
	Percentile 95	5·10^−6^
	Percentile 99	5·10^−6^
3. Logistic regression		1·10^−5^
4. Ranking		1·10^−6^

We focused on the *Ranking* and *Representation in corridors* methods and calculated the mean validation index for the points belonging to each path to appreciate the relevance of paths’ length in the results (Tables S7 and S8 in supplementary material). We observed that points of long paths yielded worse results than those of shorter paths across all *θ* values. We obtained a Pearson’s correlation coefficient between path length and the validation result of − 0.20 for the *Ranking* method and − 0.88, − 0.75, − 0.52, and − 0.30 for the *Representation in corridors* (percentiles 85, 90, 95, and 99 respectively). Additionally, the results of *Ranking* suggest that points of long paths had a better ranking position for small *θ*s, while points of short paths were better fitted with large *θ*s. The percentage of GPS points within the corridors was small for all percentiles and *θs* (Table S4 in supplementary material). However, all connectivity surfaces had a ranking percentile over 50 of the used points as compared to the random available points for all *θs* (Table S6 in supplementary material), which indicates that every connectivity surface predicted the observed lynx movements better than a random surface.

**Fig. 5:**
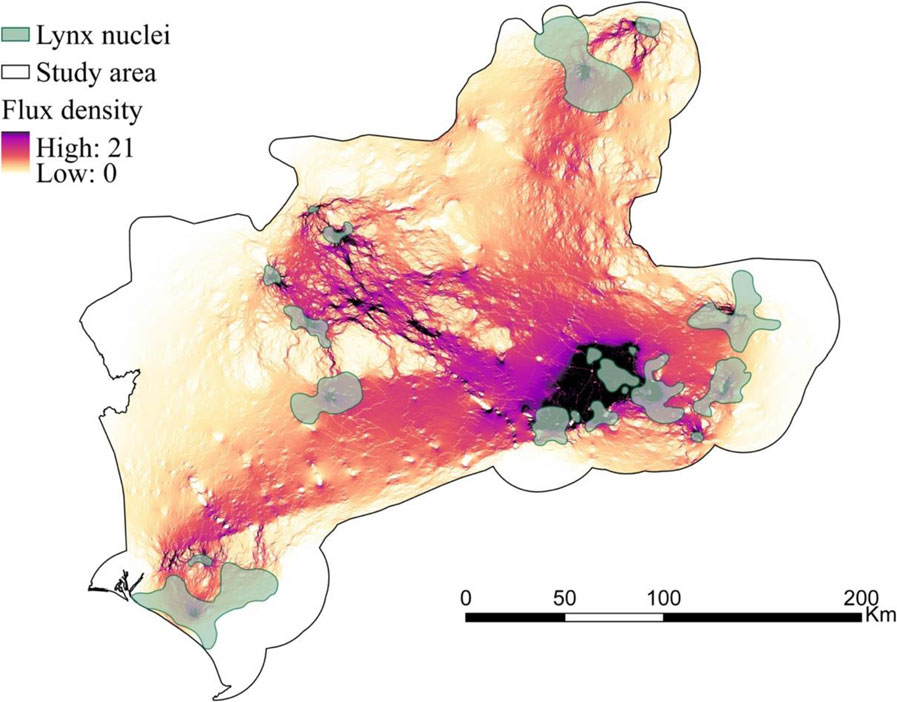
Map of flux between population nuclei for *θ* = 5·10^−6^

We assessed inter-nuclei connectivity and delineated corridors within the two traditional (LCP and circuit theory) and RSP frameworks. The optimized flux density surface (calculated with RSP and *θ* = 5 · 10^−6^, the optimal *θ* from the *Representation in corridors* validation method) can be found in Fig. 5. The corridor networks (i.e. 10% of pixels with higher flux density) corresponding to the other optimal *θ*s were very similar and shared a large amount of area with the optimized corridor network (Table 4). *Brownian bridge* was the validation method whose corridors shared less area with the optimized corridors. The corridor networks corresponding to the two extreme randomness levels (*θ*=0 and 0.01) matched to a lesser degree with the optimized corridors.

**Table 4:** Percentage of shared area between the corridor network delineated from the optimal randomness level (*θ*) indicated by the *Representation in corridors* validation method (5·10^− 6^), and that from every other optimal *θ* and connectivity modeling approach considered

Connectivity approach	Validation method	***θ***	Shared area (%)
RSP	–	0.01	56.04
RSP	*Brownian bridge*	1·10^−4^	78.66
RSP	*Resource selection function*	1·10^−5^	94.54
RSP	*Representation in corridors*	5·10^−6^	100
RSP	*Ranking*	1·10^−6^	91.22
RSP	–	0	77.24
Circuit theory	–	–	65.15
LCP	–	–	42.08

**Fig. 6:**
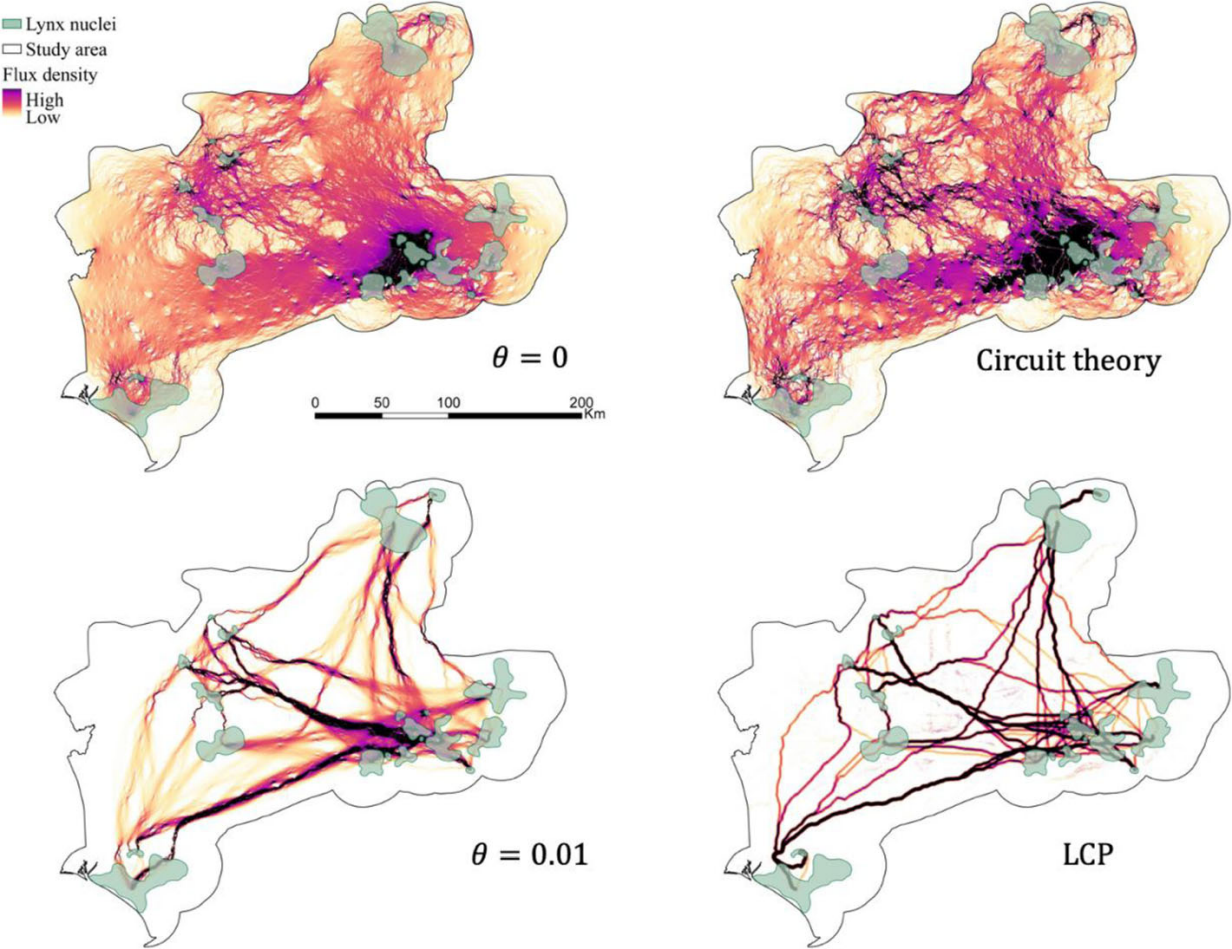
Inter-nuclei flux density calculated with Randomized Shortest Path (RSP) for the extreme *θ*s (0 and 0.01) and with the two traditional approaches: circuit theory and least-cost path (LCP)

**Fig. 7:**
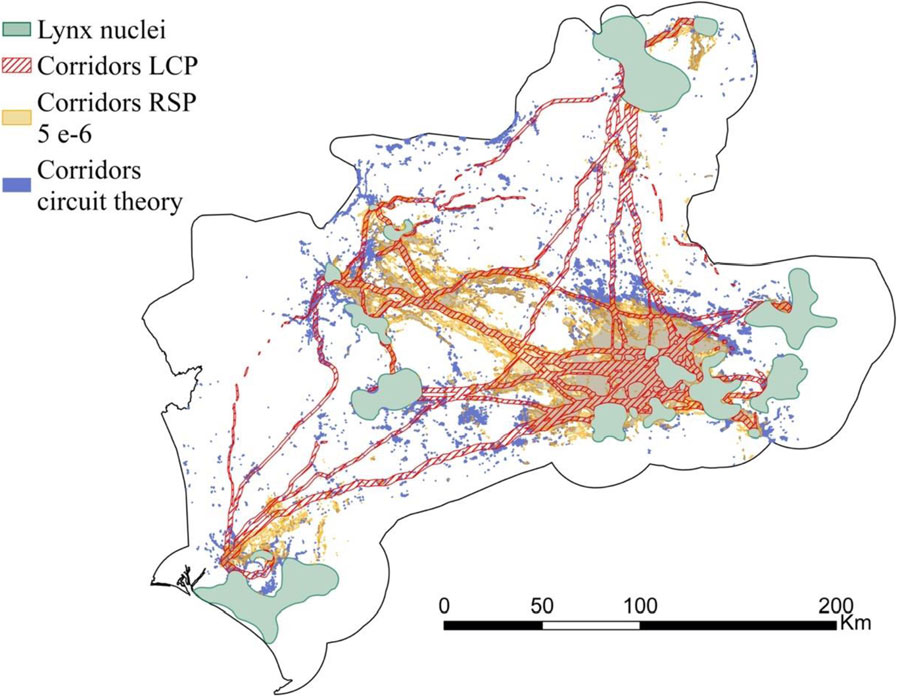
Corridors delineated from LCP (least-cost path), RSP (Randomized Shortest Path for *θ* = 5·10^−6^), and circuit theory

The inter-nuclei flux density for the extreme *θ*s and the two traditional approaches are shown in Fig. 6. The connectivity surfaces from the circuit theory approach and from RSP with *θ* = 0 were visually very similar and highly correlated (Pearson’s correlation coefficient = 0.83). There was also a high similarity between the connectivity surfaces calculated with LCP and the deterministic extreme of RSP (*θ* = 0.01). However, LCP produced more and narrower areas of movement concentration (high flux density). These two surfaces (LCP and RSP with *θ* = 0.01) had a Pearson’s correlation coefficient of 0.5. The subsequent delineation of corridors noticeably varied between the optimized RSP and the traditional approaches (Fig. 7). A percentage of 35 and 58% of the corridors corresponding to *θ* = 5 · 10^−6^ did not match with corridors delineated with circuit theory and LCP respectively (Table 4). Deterministic movements (LCP) generated thinner corridors, while random movements (circuit theory) produced fewer and broader corridors, leaving some pairs of nuclei with weak connections or completely disconnected.

## Discussion

### Lynx habitat selection for movement: the importance of the behavioral state

To assess habitat selection while moving, we considered not only the habitat selected and available by individuals but also the behavioral state of lynxes. Obtaining and modeling this information may require an additional effort compared to the most common means to obtain resistance to movement surfaces [3, 9, 65]. However, this additional effort may lead to more realistic models and thus, a thorough understanding of the relationship between the species and the environmental variables. Habitat modeling results were generally in accordance with previous studies [15, 19] about the effect of the different land cover classes on lynx habitat selection. The most preferred habitat for movement was shrublands [49, 66]. Additionally, our results showed the plasticity in habitat selection by lynxes [15, 66] for exploratory movements [19] as they select a great variety of land uses and covers for moving, including some extensive agricultural lands (i.e. olive groves). This exploratory plasticity is not exclusive to lynxes as it has been observed in other species [14, 17, 26, 67].

### Deterministic, random, or in between?

This study shows the relevance of the randomness level of species movements to wildlife connectivity studies [29, 35, 36]. Our results indicated that, for lynxes, intermediate values of *θ* outperformed the extreme values (totally random and deterministic movements) according to every validation methodology here considered (Table 3). Thus, it seems that lynxes might have some previous knowledge of the surrounding landscape and the best routes to their destinations, although they move with a certain degree of randomness, sacrificing route optimization for exploring the environment [29]. Additionally, we presented a comparative research to assess the differences and similarities between RSP and traditional approaches. RSP with extreme *θ* values produced very similar connectivity surfaces to those from the traditional frameworks (LCP and circuit theory, see Fig. 6). This similarity was especially strong for the random extreme. The other extreme of RSP represents a smoothed LCP that loosens the assumption of individuals’ selection of a unique and optimal path. This may explain the differences found with the LCP approach. Besides these differences, we can assume that RSP extremes are equivalent to the traditional approaches, and therefore, that RSP with intermediate values of *θ* generates more realistic predictions than traditional frameworks. However, both traditional methods are still being widely used, without a general consensus on which one represents better species movements [11, 18, 25, 27, 44]. In fact, it seems that the primacy of one or another methodology depends on the considered species and study area. In this study, the circuit theory approach outperformed the LCP approach as the results of the random extreme of *θ* matched better the observed movements (Tables S3 to S6 in supplementary material). Moreover, the amount of shared area with the optimized corridor network was higher for the circuit theory than for the LCP model (Table 4). However, the most suitable connectivity approach might change for different species or study areas, or when considering different factors such as path length or the age and gender of individuals. It is important to select the most suitable approach, as shown by the noticeable different corridor networks predicted through the different approaches (Fig. 7). For example, the corridor detected through RSP connecting the central-west and central-east group of nuclei, was not spotted through circuit theory. Conservation initiatives based on circuit theory approach may thus leave critical connectors without their necessary protection. On the other hand, the central-east to central-west corridor was spotted through LCP but in a narrower width than RSP. Meanwhile, other LCP corridors were not considered as especially important by RSP for lynx connectivity. Consequently, following traditional connectivity approaches may prompt the protection of suboptimal and seldom used areas or neglect important connectivity areas.

### Different validation methods: different results?

Several validation methodologies have been proposed [7, 11, 13, 14, 39–44], however, scientists have not reached a consensus regarding the most practical validation method. The alternative validation methodologies here considered showed different optimal levels of randomness, probably because of their different intrinsic assumptions. This emphasizes the importance of choosing the validation methods that better match our objectives. However, the subsequent delineation of corridors did not show big differences among the studied validation methods. This result revealed that the identification of conservation corridors might not be drastically affected by the chosen validation method to optimize the level of randomness. However, further research for other species or conservation areas would be necessary to assert this statement.

Here we considered *Representation in corridors* as the most practical method for management if the intention is to delineate, protect, or restore corridors, assuming that it allows the selection of the connectivity surface whose corridors would host more movements. By contrast, the other validation methods might be preferable to predict the general movement of the species and to understand how the species uses the landscape. *Brownian bridge*, *Logistic regression*, and *Ranking* methods inform about how much the connectivity model agrees with the observed data, assuming that the species would be most likely to use landscape units with the highest connectivity value. Particularly, *Logistic regression* is a refinement of other methodologies that only count the amount of connectivity in the used landscape [42]. Nevertheless, this method also compares the used with the available or attainable landscape. Likewise, the *Ranking* method quantifies how much each connectivity surface represents the observed movements as compared to the randomly expected. Moreover, it considers the percentile position of used points among random available ones. This aspect makes this methodology especially suitable when comparing different connectivity surfaces and areas with varying distributions of values and skewness. Additionally, we found the *Ranking* method particularly convenient, as it is more intuitive and easier to calculate than the *Logistic regression* method. Finally, the *Brownian bridge* validation method examines each point but also the route between fixes. This method is the most widely followed in previous studies inferring the optimal *θ*, however, the resulting model calculated with *Brownian bridge* (optimal *θ* = 1 · 10^−4^) presented the lowest percentage of observed GPS points inside its corridors when compared to the models calculated with the other validation methods (Table S4 in supplementary material). Additionally, *Brownian bridge* was the validation method whose corresponding corridors shared less area with the optimized corridors (Table 4).

### Importance of paths length

We found a larger agreement between the observed and predicted movements for short trajectories. In fact, in the *Representation in corridors* validation method we found a low overall percentage of points falling within corridors (Table S4 in supplementary material) but especially for long paths (Table S7 in supplementary material). Long paths comprised more GPS points than short paths and therefore had a larger impact on and reduced the overall results of the *Representation in corridors* validation method. Additionally, short tracks were better represented by larger *θ*s (more deterministic movements) than longer paths. This might suggest that lynxes move with different levels of randomness depending on the distance to the destination point. Concretely, individuals may be more habituated to the landscape in short movements and therefore, could identify more easily the best route. On the other hand, in long movements, individuals could be unfamiliar with the landscape, leading to more exploratory and random movements. However, further research is needed to understand the effects of different path lengths and time lapses on individuals’ movements. Additionally, the effect of other factors (e.g., seasonality, age, sex) on the level of randomness may be worthy of study.

## Conclusions

Assessing animal connectivity is a complex and challenging process that carries important implications when guiding decisions for landscape management. It involves multiple factors such as the location and behavioral state of individuals, habitat preference while moving, dispersal capacities, and randomness level of the species movements. This paper has sought to cast some light on the consideration of these factors to generate improved, reliable, and functional connectivity models. The proper consideration of these issues may contribute to successful conservation and restoration actions based on steadily improving ecological understanding of animal movements.

Additionally, the empirical validation here conducted has contributed to examine uncertainties and fill the gap of comparative research in connectivity assessments. We showed that the alternative validation methodologies here considered are based on different assumptions that led to different optimal randomness levels. Therefore, it is important to choose the validation method (or a combination of them) that better matches the objectives of the case study. However, these differences among validation methods might not affect noticeably the identification of corridors, as every validation method led to very similar corridor networks. Additionally, every validation methodology agreed about the better performance of intermediate levels of randomness (from the RSP framework) than extreme values (from traditional connectivity approaches), proving that lynxes move neither in a totally random nor deterministic way.

Finally, our results showed that the distribution of corridors between lynx population nuclei varied according to the level of randomness. Following traditional connectivity approaches may thus lead to the protection and allocation of resources in suboptimal areas. On the other hand, selecting the most suitable level of randomness advances towards more reliable and functional models. In this way, applying these insights when modeling connectivity would lead to making sound and operational decisions and thus more effective and efficient management actions. The reported findings are of major importance for this particular focal species, but also have a broader impact to guide conceptually related studies.

## Supplementary Information

**Additional file 1: Fig. S1.** Conductance surface. **Fig S2.** Connectivity surfaces from seven different *θ*s: 5·10^− 7^, 1·10^− 6^, 5·10^− 6^, 5·10^− 5^, 1·10^− 4^, 5·10^− 4^, and 0.005. The values of all surfaces range from 0 to 2. **Table S3.** Results of the validation methodology *Brownian bridge*. The minimum MSE corresponds to the optimal *θ*. **Table S4.** Validation results for the *Representation in corridors* methodology: percentage of GPS points inside corridors. **Table S5.** Results of the *Logistic regression* validation methodology. The lowest AIC indicates the optimal *θ*. **Table S6.** Results for the validation *Ranking* technique. Rank position of used points when compared to available ones. **Table S7.** Percentage of points of each path that fall in the percentile 90 corridor. Pearson’s correlation coefficient (PCC) between the mean percentage of each path and the path length. **Table S8.** Mean rank of the GPS points (compared to available points) of each path for the *Ranking* validation method. Pearson’s correlation coefficient (PCC) between the mean rank of each path and the path length.

## Data Availability

The data that support the findings of this study are guarded by the “Consejería de Agricultura, Ganadería, Pesca y Desarrollo Sostenible” (CAGPDS) of the “Junta de Andalucía” but restrictions apply to the availability of these data, which were used under license for the current study, and so are not publicly available. Data are however available from CAGPDS if requested with justification of use.

## Abbreviations

PSF: Point selection functions
LCP: Least-cost path
RSP: Randomized Shortest Path
CLCS: Copernicus Land Cover Services
LCM: Land cover map
DEM: Digital elevation model
AUC: Area under the receiver operating characteristic curve
AIC: Akaike’s information criterion

## References

[1] Taylor PD, Fahrig L, Henein K, Merriam G, Taylor PD, Fahrig L (1993). Connectivity is a vital element of landscape structure. Oikos..

[2] Crooks KR, Sanjayan M. Connectivity conservation. In: Kevin R, Crooks MS, editors. Connectivity Conservation. Cambridge; 2006. p. 1–20. Available from: http://ebooks.cambridge.org/ref/id/CBO9780511754821A008. Accessed 24 Jan 2017.

[3] Correa Ayram CA, Mendoza ME, Etter A, Pérez Salicrup DR. Habitat connectivity in biodiversity conservation : a review of recent studies and applications. Prog Phys Geogr. 2015;40(1):1–32.

[4] Keeley ATH, Ackerly DD, Cameron DR, Heller NE, Huber PR, Schloss CA (2018). New concepts , models , and assessments of climate-wise connectivity New concepts , models , and assessments of climate-wise connectivity. Environ Res Lett.

[5] Gurrutxaga M, Saura S (2013). Prioritizing highway defragmentation locations for restoring landscape connectivity. Environ Conserv.

[6] de la Fuente B, Mateo-Sánchez MC, Rodríguez G, Gastón A (2018). Pérez de Ayala R, Colomina-Pérez D, et al. Natura 2000 sites, public forests and riparian corridors: the connectivity backbone of forest green infrastructure. Land Use Policy.

[7] McClure ML, Hansen AJ, Inman RM (2016). Connecting models to movements: testing connectivity model predictions against empirical migration and dispersal data. Landsc Ecol.

[8] Laliberté J, St-Laurent MH. Validation of functional connectivity modeling: The Achilles’ heel of landscape connectivity mapping. Landsc Urban Plan. 2020;202(June):1–11.

[9] Zeller KA, McGarigal K, Whiteley AR (2012). Estimating landscape resistance to movement: a review. Landsc Ecol.

[10] Manly BF, McDonald L, Thomas DL, McDonald TL, Erickson WP. In: Springer Science & Business Media, editor. Resource selection by animals: statistical design and analysis for field studies: USA: Springer Science & Business Media; 2007.

[11] Pullinger MG, Johnson CJ (2010). Maintaining or restoring connectivity of modified landscapes: evaluating the least-cost path model with multiple sources of ecological information. Landsc Ecol.

[12] Zeller KA, McGarigal K, Beier P, Cushman SA, Vickers TW, Boyce WM (2014). Sensitivity of landscape resistance estimates based on point selection functions to scale and behavioral state: pumas as a case study. Landsc Ecol.

[13] Zeller KA, Jennings MK, Vickers TW, Ernest HB, Cushman SA, Boyce WM (2018). Are all data types and connectivity models created equal? Validating common connectivity approaches with dispersal data. Divers Distrib.

[14] Abrahms B, Sawyer SC, Jordan NR, McNutt JW, Wilson AM, Brashares JS (2017). Does wildlife resource selection accurately inform corridor conservation?. J Appl Ecol.

[15] Gastón A, Blázquez-Cabrera S, Ciudad C, Mateo Sánchez MC, Simón MA, Saura S (2019). The role of forest canopy cover in habitat selection: insights from the Iberian lynx. Eur J Wildl Res.

[16] Tischendorf L, Fahrig L (2000). On the usage and measurement of landscape connectivity. Oikos..

[17] Elliot NB, Cushman SA, Macdonald DW, Loveridge AJ (2014). The devil is in the dispersers: predictions of landscape connectivity change with demography. J Appl Ecol.

[18] Maiorano L, Boitani L, Chiaverini L, Ciucci P (2017). Uncertainties in the identification of potential dispersal corridors: the importance of behaviour, sex, and algorithm. Basic Appl Ecol.

[19] Gastón A, Blázquez-Cabrera S, Garrote G, Mateo-Sánchez MC, Beier P, Simón MA, Saura S (2016). Response to agriculture by a woodland species depends on cover type and behavioural state: insights from resident and dispersing Iberian lynx. J Appl Ecol.

[20] Cushman SA, McKelvey KS, Schwartz MK (2009). Use of empirically derived source-destination models to map regional conservation corridors. Conserv Biol.

[21] Adriaensen F, Chardon JP, De Blust G, Swinnen E, Villalba S, Gulinck H (2003). The application of “least-cost” modelling as a functional landscape model. Landsc Urban Plan.

[22] Etherington TR (2016). Least-cost modelling and landscape ecology: concepts, applications, and opportunities. Curr Landsc Ecol Reports.

[23] Fahrig L (2007). Non-optimal animal movement in human-altered landscapes. Funct Ecol.

[24] McRae BH, Dickson BG, Keitt TH, Shah VB (2008). Using circuit theory to model conectivity in ecology, evolution, and conservatin. Ecology..

[25] Castilho CS, Hackbart VCS, Pivello VR, dos Santos RF (2015). Evaluating landscape connectivity for Puma concolor and Panthera onca among Atlantic Forest protected areas. Environ Manag.

[26] Mateo Sánchez MC, Balkenhol N, Cushman S, Pérez T, Domínguez A, Saura S (2015). Estimating effective landscape distances and movement corridors : comparison of habitat and genetic data. Ecosphere..

[27] Dilts TE, Weisberg PJ, Leitner P, Matocq MD, Inman RD, Nussear KE, Esque TC (2016). Multiscale connectivity and graph theory highlight critical areas for conservation under climate change. Ecol Appl.

[28] Coulon AA, Aben J, Palmer SF, Stevens VM, Callens T, Strubbe D (2015). A stochastic movement simulator improves estimates of landscape connectivity. Ecology..

[29] Panzacchi M, Van Moorter B, Strand O, Saerens M, Kivimäki I, St. Clair CC (2016). Predicting the continuum between corridors and barriers to animal movements using step selection functions and randomized shortest paths. J Anim Ecol.

[30] Van Moorter B, Kivimäki I, Panzacchi M, Saerens M. Defining and quantifying effective connectivity of landscapes for species’ movements. Ecography (Cop). 2021;44(6):1–15.

[31] Saerens M, Achbany Y, Fouss F, Yen L (2009). Randomized shortest-path problems: two related models. Neural Comput.

[32] Kivimäki I, Shimbo M, Saerens M (2014). Developments in the theory of randomized shortest paths with a comparison of graph node distances. Physica A.

[33] Taylor PD, Fahrig L, With KA. Landscape connectivity: a return to the basics. In: Connectivity conservation. Cambridge University Press: Cambridge; 2006.

[34] Rudnick DA, Ryan SJ, Beier P, Cushman SA, Dieffenbach F, Epps CW (2012). The role of landscape connectivity in planning and implementing conservation and restoration priorities. Issues Ecol.

[35] Fullman TJ, Joly K, Ackerman A (2017). Effects of environmental features and sport hunting on caribou migration in northwestern Alaska. Mov Ecol.

[36] Long JA (2019). Estimating wildlife utilization distributions using randomized shortest paths. Landsc Ecol.

[37] Brennan A, Hanks EM, Merkle JA, Cole EK, Dewey SR, Courtemanch AB (2018). Examining speed versus selection in connectivity models using elk migration as an example. Landsc Ecol.

[38] Peck CP, VanManen FT, Costello CM, Haroldson MA, Landenburger LA, Roberts LL, et al. Potential paths for male-mediated gene flow to and from an isolated grizzly bear population. Ecosphere. 2017;8(10):e01969.

[39] Driezen K, Adriaensen F, Rondinini C, Doncaster CP, Matthysen E (2007). Evaluating least-cost model predictions with empirical dispersal data: a case-study using radiotracking data of hedgehogs (Erinaceus europaeus). Ecol Model.

[40] Poor EE, Loucks C, Jakes A, Urban DL. Comparing habitat suitability and connectivity modeling methods for conserving pronghorn migrations. PLoS One. 2012;7(11):e49390. 10.1371/journal.pone.0049390 PMC3500376 23166656

[41] Stevenson CD, Ferryman M, Nevin OT, Ramsey AD, Bailey S, Watts K (2013). Using GPS telemetry to validate least-cost modeling of gray squirrel (Sciurus carolinensis) movement within a fragmented landscape. Ecol Evol.

[42] Trainor AM, Walters JR, Morris WF, Sexton J, Moody A (2013). Empirical estimation of dispersal resistance surfaces : a case study with red-cockaded woodpeckers. Landsc Ecol.

[43] Cushman SA, Lewis JS, Landguth EL (2014). Why did the bear cross the road? Comparing the performance of multiple resistance surfaces and connectivity modeling methods. Diversity..

[44] Osipova L, Okello MM, Njumbi SJ, Ngene S, Western D, Hayward MW (2019). Validating movement corridors for African elephants predicted from resistance-based landscape connectivity models. Landsc Ecol.

[45] Rodríguez A, Calzada J (2015). The IUCN red list of threatened species.

[46] Simón MA, Gil-Sánchez JM, Ruiz G, Garrote G, Mccain EB, Fernández L (2012). Reverse of the decline of the endangered Iberian Lynx. Conserv Biol.

[47] Rodríguez A, Delibes M (1992). Current range and status of the Iberian lynx Felis pardina Temminck, 1824 in Spain. Biol Conserv.

[48] Life+IBERLINCE. Available from: http://www.iberlince.eu/. Cited 2021 Apr 20

[49] Palomares F, Delibes M, Ferreras P, Fedriani JM, Calzada J, Revilla E (2000). Iberian lynx in a fragmented landscape: Predispersal, dispersal, and postdispersal habitats. Conserv Biol.

[50] Palomares F (2001). Vegetation structure and prey abundance requirements of the Iberian lynx: implications for the design of reserves and corridors. J Appl Ecol.

[51] Blazquez-Cabrera S, Gastón A, Beier P, Garrote G, Simón MÁ, Saura S (2016). Influence of separating home range and dispersal movements on characterizing corridors and effective distances. Landsc Ecol.

[52] Getz WM, Fortmann-Roe S, Cross PC, Lyons AJ, Ryan SJ, Wilmers CC (2007). LoCoH: Nonparameteric kernel methods for constructing home ranges and utilization distributions. PLoS One.

[53] Huck M, Davison J, Roper TJ (2008). Comparison of two sampling protocols and four home-range estimators using radio-tracking data from urban badgers Meles meles. Wildl Biol.

[54] Podgórski T, Baś G, Jędrzejewska B, Sönnichsen L, Śnieżko S, Jędrzejewski W, Okarma H (2013). Spatiotemporal behavioral plasticity of wild boar ( Sus scrofa ) under contrasting conditions of human pressure : primeval forest and metropolitan area. J Mammal.

[55] Getz WM, Wilmers CC (2004). A local nearest-neighbor convex-hull construction of home ranges and utilization distributions. Ecography (Cop).

[56] Ministerio de Fomento (2015). Plan Nacional de Ortofotografía Aérea.

[57] Ribatet M (2011). POT: generalized Pareto distribution and peaks over threshold.

[58] Zeller KA, McGarigal K, Cushman SA, Beier P, Vickers TW, Boyce WM (2016). Using step and path selection functions for estimating resistance to movement: pumas as a case study. Landsc Ecol.

[59] Pearce J, Ferrier S (2000). Evaluating the predictive performance of habitat models developed using logistic regression. Ecol Model.

[60] Roberts DR, Bahn V, Ciuti S, Boyce MS, Elith J, Guillera-Arroita G, Hauenstein S, Lahoz-Monfort JJ, Schröder B, Thuiller W, Warton DI, Wintle BA, Hartig F, Dormann CF (2017). Cross-validation strategies for data with temporal, spatial, hierarchical, or phylogenetic structure. Ecography (Cop).

[61] van Etten J. R package gdistance: Distances and routes on geographical grids. J Stat Softw. 2017;76(1):1–21.

[62] Horne JS, Garton EO, Krone SM, Lewis JS (2007). Analyzing animal movements using Brownian bridges. Ecology..

[63] Landguth EL, Hand BK, Glassy J, Cushman SA, Sawaya MA (2012). UNICOR : a species connectivity and corridor network simulator. Ecography (Cop).

[64] Anantharaman R, Hall K, Shah V, Edelman A. Circuitscape in julia: High performance connectivity modelling to support conservation decisions. Proc JuliaCon Conf. 2020;1(1). 10.21105/jcon.00058.

[65] Cushman SA, Mcrae B, Adriaensen F, Beier P, Shirley M, Zeller K (2013). Biological corridors and connectivity. Key Topics in Conservation Biology 2.

[66] Garrote G, Bueno JF, Ruiz M, de Lillo S, Martin JM, Moral M, Simón MA (2020). Breaking barriers: Iberian Lynx Lynx pardinus Temminck, 1827 (Mammalia: Carnivora: Felidae) colonizing olive groves Germán. J Threat Taxa.

[67] Keeley ATH, Beier P, Gagnon JW (2016). Estimating landscape resistance from habitat suitability: effects of data source and nonlinearities. Landsc Ecol.

