# Supplementary material for: Deterministic, random, or in between? Inferring the randomness level of wildlife movements
Source: Mov Ecol. 2021 Jun 29;9:33. doi: 10.1186/s40462-021-00273-7 (PMC8244215; doi:10.1186/s40462-021-00273-7)
Supplement: Supplementary file 1 — Additional file 1: Fig. S1. Conductance surface. Fig S2. Connectivity surfaces from seven different θs: 5·10− 7, 1·10− 6, 5·10− 6, 5·10− 5, 1·10− 4, 5·10− 4, and 0.005. The values of all surfaces range from 0 to 2. Table S3. Results of the validation methodology Brownian bridge. The minimum MSE corresponds to the optimal θ. Table S4. Validation results for the Representation in corridors methodology: percentage of GPS points inside corridors. Table S5. Results of the Logistic regression validation methodology. The lowest AIC indicates the optimal θ. Table S6. Results for the validation Ranking technique. Rank position of used points when compared to available ones. Table S7. Percentage of points of each path that fall in the percentile 90 corridor. Pearson’s correlation coefficient (PCC) between the mean percentage of each path and the path length. Table S8. Mean rank of the GPS points (compared to available points) of each path for the Ranking validation method. Pearson’s correlation coefficient (PCC) between the mean rank of each path and the path length. [file 40462_2021_273_MOESM1_ESM.docx]

**Supplementary material: Deterministic, random, or in between? Inferring the randomness level of wildlife movements**

Teresa Goicolea, Aitor Gastón, Pablo Cisneros-Araujo, Juan Ignacio García-Viñas, M Cruz Mateo-Sánchez.

ETSI Montes, Forestal y del Medio Natural, Universidad Politécnica de Madrid, Ciudad Universitaria s/n, 28040, Madrid, Spain.

Corresponding author: Teresa Goicolea, ORCID: 0000-0002-4069-6001

e-mail address: t.goicolea@gmail.com; Telephone number: +34 699927115


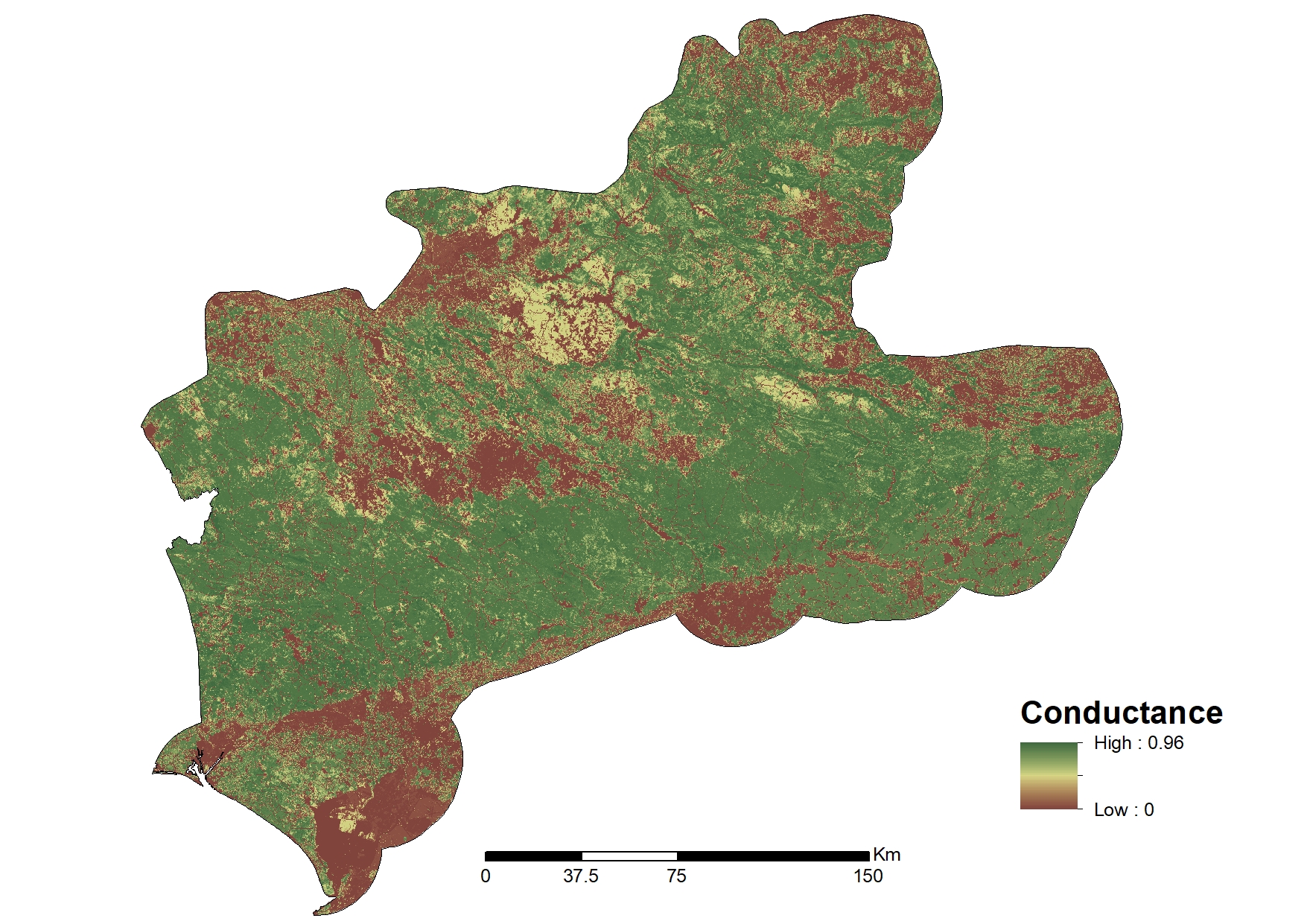


**Fig. S1** Conductance surface


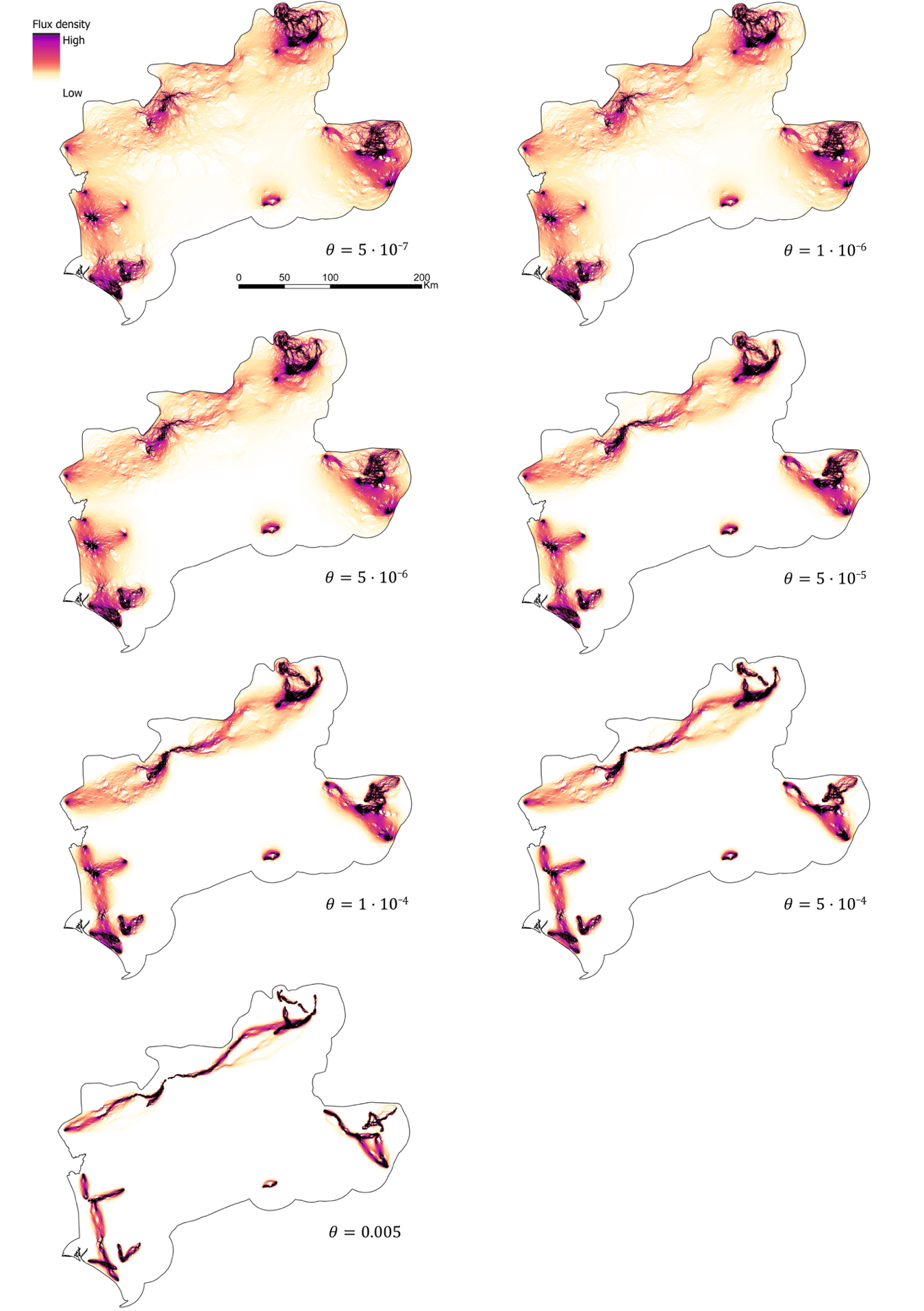


**Fig S2** Connectivity surfaces from seven different $\theta$s: 5·10^-7^, 1·10^-6^, 5·10^-6^, 5·10^-5^, 1·10^-4^, 5·10^-4^, and 0.005. The values of all surfaces range from 0 to 2.

**Table S3** Results of the validation methodology *Brownian bridge*. The minimum MSE corresponds to the optimal $\theta$

| $\boldsymbol{\theta}$ | **MSE** |
| --- | --- |
| 0 | 6.06E-12 |
| 5.E-07 | 6.02E-12 |
| 1.E-06 | 6.01E-12 |
| 5.E-06 | 5.99E-12 |
| 1.E-05 | 5.98E-12 |
| 5.E-05 | 5.96E-12 |
| 1.E-04 | 5.96E-12 |
| 5.E-04 | 5.99E-12 |
| 0.001 | 6.03E-12 |
| 0.005 | 6.23E-12 |
| 0.01 | 6.36E-12 |

**Table S4.** Validation results for the *Representation in corridors* methodology: percentage of GPS points inside corridors.

|  | **Point level** | | | |
| --- | --- | --- | --- | --- |
|  | **Percentile 85** | **Percentile 90** | **Percentile 95** | **Percentile 99** |
| $\boldsymbol{\theta}$ |  |  |  |  |
| 0 | 44.83 | 39.93 | 30.58 | 12.21 |
| 5·E-07 | 45.82 | 41.64 | 31.57 | 12.38 |
| 1·E-06 | 46.15 | 41.86 | 31.79 | 12.65 |
| 5·E-06 | 46.53 | 42.30 | 32.23 | 13.37 |
| 1·E-05 | 46.15 | 41.91 | 32.23 | 13.26 |
| 5·E-05 | 45.05 | 41.14 | 31.57 | 13.15 |
| 1·E-04 | 44.72 | 40.54 | 31.57 | 13.04 |
| 5E-04 | 43.12 | 39.22 | 30.25 | 12.87 |
| 0.001 | 43.12 | 37.90 | 29.65 | 12.93 |
| 0.005 | 42.57 | 36.41 | 28.11 | 10.56 |
| 0.01 | 42.57 | 35.86 | 27.61 | 10.12 |

**Table S5** Results of the *Logistic regression* validation methodology. The lowest AIC indicates the optimal $\theta$.

|  |  | |
| --- | --- | --- |
| $\boldsymbol{\theta}$ | **AIC** |  |
| 0 | 2278.34 | |
| 5.E-07 | 2267.03 | |
| 1.E-06 | 2264.36 | |
| 5.E-06 | 2258.21 | |
| 1.E-05 | 2256.88 | |
| 5.E-05 | 2257.97 | |
| 1.E-04 | 2261.67 | |
| 5.E-04 | 2286.70 | |
| 0.001 | 2304.96 | |
| 0.005 | 2367.95 | |
| 0.01 | 2397.91 | |

**Table S6** Results for the validation *Ranking* technique. Rank position of used points when compared to available ones.

| $\boldsymbol{\theta}$ | **Mean percentile** | **Standard deviation** |
| --- | --- | --- |
| 0 | 61.58 | 24.52 |
| 5.E-07 | 61.63 | 24.54 |
| 1.E-06 | 61.63 | 24.53 |
| 5.E-06 | 61.56 | 24.33 |
| 1.E-05 | 61.53 | 24.14 |
| 5.E-05 | 61.32 | 23.10 |
| 1.E-04 | 61.09 | 22.44 |
| 5.E-04 | 60.49 | 20.77 |
| 0.001 | 60.07 | 20.22 |
| 0.005 | 59.33 | 19.28 |
| 0.01 | 59.13 | 19.06 |

**Table S7** Percentage of points of each path that fall in the percentile 90 corridor. Pearson’s correlation coefficient (PCC) between the mean percentage of each path and the path length.

|  | $\boldsymbol{\theta}$ | | | | | | | | | | | | |  | |  |
| --- | --- | --- | --- | --- | --- | --- | --- | --- | --- | --- | --- | --- | --- | --- | --- | --- |
| **Path** | **0** | **5·E-07** | **1·E-06** | **5·E-06** | **1·E-05** | **5·E-05** | **1·E-04** | **5·E-04** | **0.001** | **0.005** | **0.01** | **Mean of each path** | **Path length (m)** | |  |  |
| 1 | 65.52 | 68.97 | 68.97 | 68.97 | 68.97 | 68.97 | 68.97 | 68.97 | 68.97 | 68.97 | 68.97 | 68.65 | 15390 | |  |  |
| 2 | 47.62 | 61.90 | 61.90 | 66.67 | 66.67 | 66.67 | 66.67 | 66.67 | 61.90 | 52.38 | 52.38 | 61.04 | 15913 | |  |  |
| 3 | 66.67 | 66.67 | 66.67 | 66.67 | 66.67 | 66.67 | 66.67 | 63.64 | 63.64 | 60.61 | 60.61 | 65.01 | 16899 | |  |  |
| 4 | 60.00 | 60.00 | 60.00 | 70.00 | 70.00 | 70.00 | 60.00 | 50.00 | 50.00 | 40.00 | 40.00 | 57.27 | 18275 | |  |  |
| 5 | 88.89 | 88.89 | 88.89 | 88.89 | 88.89 | 88.89 | 88.89 | 88.89 | 88.89 | 77.78 | 66.67 | 85.86 | 18283 | |  |  |
| 6 | 57.14 | 66.67 | 66.67 | 71.43 | 71.43 | 71.43 | 76.19 | 76.19 | 76.19 | 71.43 | 71.43 | 70.56 | 22383 | |  |  |
| 7 | 72.73 | 72.73 | 72.73 | 72.73 | 72.73 | 63.64 | 63.64 | 63.64 | 63.64 | 63.64 | 54.55 | 66.94 | 22460 | |  |  |
| 8 | 75.00 | 75.00 | 75.00 | 75.00 | 75.00 | 75.00 | 75.00 | 68.75 | 68.75 | 62.50 | 62.50 | 71.59 | 25434 | |  |  |
| 9 | 75.00 | 75.00 | 75.00 | 75.00 | 75.00 | 75.00 | 75.00 | 87.50 | 87.50 | 87.50 | 87.50 | 79.55 | 26430 | |  |  |
| 10 | 70.59 | 70.59 | 70.59 | 70.59 | 70.59 | 70.59 | 70.59 | 64.71 | 58.82 | 35.29 | 35.29 | 62.57 | 26653 | |  |  |
| 11 | 65.00 | 65.00 | 65.00 | 65.00 | 65.00 | 55.00 | 55.00 | 55.00 | 55.00 | 55.00 | 55.00 | 59.55 | 29209 | |  |  |
| 12 | 44.44 | 50.00 | 55.56 | 61.11 | 61.11 | 72.22 | 66.67 | 61.11 | 55.56 | 61.11 | 61.11 | 59.09 | 32881 | |  |  |
| 13 | 65.12 | 72.09 | 72.09 | 72.09 | 72.09 | 72.09 | 72.09 | 72.09 | 69.77 | 60.47 | 55.81 | 68.71 | 33017 | |  |  |
| 14 | 66.67 | 66.67 | 66.67 | 64.81 | 64.81 | 61.11 | 55.56 | 44.44 | 42.59 | 42.59 | 40.74 | 56.06 | 36499 | |  |  |
| 15 | 61.11 | 61.11 | 61.11 | 61.11 | 59.26 | 57.41 | 53.70 | 40.74 | 27.78 | 18.52 | 18.52 | 47.31 | 37515 | |  |  |
| 16 | 50.00 | 50.00 | 50.00 | 57.14 | 50.00 | 57.14 | 57.14 | 50.00 | 35.71 | 42.86 | 42.86 | 49.35 | 37776 | |  |  |
| 17 | 66.67 | 76.19 | 76.19 | 76.19 | 76.19 | 76.19 | 76.19 | 76.19 | 71.43 | 47.62 | 42.86 | 69.26 | 40581 | |  |  |
| 18 | 50.85 | 54.24 | 54.24 | 55.93 | 55.93 | 55.93 | 55.93 | 55.93 | 55.93 | 54.24 | 52.54 | 54.70 | 42044 | |  |  |
| 19 | 63.16 | 73.68 | 73.68 | 78.95 | 78.95 | 78.95 | 78.95 | 73.68 | 73.68 | 63.16 | 63.16 | 72.73 | 42650 | |  |  |
| 20 | 47.83 | 47.83 | 47.83 | 52.17 | 52.17 | 65.22 | 65.22 | 65.22 | 65.22 | 82.61 | 82.61 | 61.26 | 42676 | |  |  |
| 21 | 58.82 | 52.94 | 52.94 | 47.06 | 47.06 | 47.06 | 47.06 | 41.18 | 41.18 | 35.29 | 35.29 | 45.99 | 44892 | |  |  |
| 22 | 73.08 | 73.08 | 76.92 | 76.92 | 76.92 | 76.92 | 76.92 | 69.23 | 69.23 | 69.23 | 65.38 | 73.08 | 45718 | |  |  |
| 23 | 34.78 | 34.78 | 34.78 | 43.48 | 43.48 | 47.83 | 52.17 | 56.52 | 60.87 | 65.22 | 65.22 | 49.01 | 47754 | |  |  |
| 24 | 65.22 | 78.26 | 78.26 | 82.61 | 86.96 | 86.96 | 86.96 | 82.61 | 73.91 | 43.48 | 43.48 | 73.52 | 51103 | |  |  |
| 25 | 60.00 | 62.50 | 62.50 | 62.50 | 62.50 | 62.50 | 55.00 | 42.50 | 42.50 | 42.50 | 42.50 | 54.32 | 53033 | |  |  |
| 26 | 50.00 | 53.85 | 53.85 | 53.85 | 53.85 | 53.85 | 53.85 | 53.85 | 53.85 | 50.00 | 50.00 | 52.80 | 54478 | |  |  |
| 27 | 63.64 | 65.91 | 68.18 | 68.18 | 68.18 | 65.91 | 68.18 | 70.45 | 75.00 | 70.45 | 70.45 | 68.60 | 57333 | |  |  |
| 28 | 53.45 | 53.45 | 53.45 | 56.90 | 56.90 | 56.90 | 56.90 | 56.90 | 55.17 | 55.17 | 55.17 | 55.49 | 58865 | |  |  |
| 29 | 85.42 | 85.42 | 85.42 | 85.42 | 85.42 | 85.42 | 85.42 | 85.42 | 85.42 | 81.25 | 81.25 | 84.66 | 65798 | |  |  |
| 30 | 64.77 | 63.64 | 63.64 | 48.86 | 47.73 | 31.82 | 23.86 | 22.73 | 22.73 | 22.73 | 21.59 | 39.46 | 111408 | |  |  |
| 31 | 43.82 | 47.19 | 47.19 | 47.19 | 46.07 | 44.94 | 43.82 | 40.45 | 39.33 | 40.45 | 40.45 | 43.72 | 121563 | |  |  |
| 32 | 53.03 | 55.30 | 55.30 | 56.82 | 53.79 | 51.52 | 49.24 | 46.21 | 36.36 | 29.55 | 29.55 | 46.97 | 147812 | |  |  |
| 33 | 5.59 | 7.24 | 7.89 | 8.88 | 9.21 | 9.87 | 10.20 | 11.18 | 12.50 | 16.45 | 17.11 | 10.56 | 292057 | |  |  |
| 34 | 15.50 | 15.50 | 15.25 | 16.50 | 16.25 | 16.75 | 18.25 | 20.00 | 20.25 | 20.75 | 20.00 | 17.73 | 431359 | |  |  |
| **Mean of each** $\boldsymbol{\theta}$ | 58.44 | 60.95 | 61.30 | 62.52 | 62.23 | 61.95 | 61.06 | 58.61 | 56.74 | 52.67 | 51.55 | **PCC** | **-0.75** | |  |  |

**Table S8** Mean rank of the GPS points (compared to available points) of each path for the *Ranking* validation method. Pearson’s correlation coefficient (PCC) between the mean rank of each path and the path length.

|  | | $\boldsymbol{\theta}$ | | | | | | | | | | |  | |  | |  |
| --- | --- | --- | --- | --- | --- | --- | --- | --- | --- | --- | --- | --- | --- | --- | --- | --- | --- |
| **Path** | **0** | | **5·E-07** | **1·E-06** | **5·E-06** | **1·E-05** | **5·E-05** | **1·E-04** | **5·E-04** | **0.001** | **0.005** | **0.01** | | **Mean of each path** | | **Path length (m)** | |
| 1 | 74.91 | | 75.53 | 75.83 | 76.83 | 77.47 | 79.66 | 80.69 | 82.13 | 82.22 | 80.67 | 78.63 | | 78.60 | | 15390.36 | |
| 2 | 68.67 | | 70.92 | 71.50 | 73.65 | 75.08 | 78.50 | 79.71 | 81.63 | 81.92 | 81.38 | 80.83 | | 76.71 | | 15912.92 | |
| 3 | 76.01 | | 76.48 | 76.61 | 77.18 | 77.47 | 78.22 | 78.35 | 77.75 | 77.18 | 76.32 | 76.06 | | 77.06 | | 16899.22 | |
| 4 | 51.48 | | 52.21 | 52.33 | 52.73 | 52.78 | 52.76 | 53.10 | 52.82 | 52.56 | 50.77 | 49.07 | | 52.05 | | 18274.74 | |
| 5 | 42.12 | | 42.29 | 42.26 | 42.61 | 42.83 | 43.90 | 44.90 | 48.35 | 49.61 | 50.20 | 49.27 | | 45.30 | | 18282.80 | |
| 6 | 68.97 | | 70.30 | 70.45 | 70.78 | 70.92 | 71.11 | 71.16 | 71.24 | 71.13 | 70.87 | 70.75 | | 70.70 | | 22382.65 | |
| 7 | 60.59 | | 59.90 | 59.73 | 59.02 | 58.84 | 58.79 | 59.07 | 59.09 | 58.71 | 53.95 | 51.65 | | 58.12 | | 22459.56 | |
| 8 | 59.94 | | 60.68 | 60.84 | 61.65 | 62.14 | 63.95 | 64.90 | 67.17 | 67.70 | 66.42 | 65.13 | | 63.68 | | 25434.28 | |
| 9 | 54.71 | | 55.35 | 55.78 | 56.07 | 56.36 | 59.75 | 61.72 | 64.61 | 66.31 | 69.94 | 71.38 | | 61.09 | | 26430.48 | |
| 10 | 73.37 | | 73.90 | 73.76 | 72.81 | 71.84 | 68.71 | 67.38 | 64.42 | 63.28 | 59.59 | 58.14 | | 67.93 | | 26653.09 | |
| 11 | 55.18 | | 55.11 | 55.08 | 55.19 | 55.33 | 56.72 | 57.68 | 60.94 | 62.59 | 65.99 | 66.83 | | 58.79 | | 29209.48 | |
| 12 | 49.43 | | 49.48 | 49.57 | 50.35 | 50.68 | 52.84 | 53.75 | 54.32 | 54.73 | 55.12 | 55.30 | | 52.32 | | 32881.11 | |
| 13 | 61.68 | | 62.59 | 62.94 | 64.13 | 64.75 | 66.17 | 66.48 | 66.37 | 66.29 | 67.98 | 67.88 | | 65.21 | | 33016.94 | |
| 14 | 65.60 | | 66.18 | 65.80 | 63.05 | 61.88 | 60.59 | 60.41 | 61.19 | 61.68 | 62.14 | 62.11 | | 62.78 | | 36499.09 | |
| 15 | 73.76 | | 74.78 | 75.07 | 75.96 | 76.33 | 76.38 | 75.64 | 72.68 | 71.30 | 69.80 | 69.95 | | 73.79 | | 37515.30 | |
| 16 | 48.61 | | 49.50 | 49.81 | 50.46 | 50.63 | 49.73 | 49.83 | 50.80 | 51.21 | 52.57 | 53.68 | | 50.62 | | 37776.04 | |
| 17 | 65.77 | | 66.08 | 66.14 | 66.20 | 66.18 | 65.32 | 64.34 | 62.95 | 62.34 | 61.60 | 60.06 | | 64.27 | | 40580.74 | |
| 18 | 77.45 | | 78.52 | 78.79 | 79.52 | 79.90 | 80.66 | 80.86 | 79.97 | 77.58 | 68.56 | 67.48 | | 77.21 | | 42044.48 | |
| 19 | 70.25 | | 70.63 | 70.70 | 71.25 | 71.69 | 72.99 | 73.53 | 73.66 | 72.74 | 69.20 | 68.20 | | 71.35 | | 42650.42 | |
| 20 | 55.24 | | 55.09 | 54.94 | 54.13 | 53.75 | 52.83 | 52.50 | 52.95 | 54.06 | 57.14 | 58.31 | | 54.63 | | 42675.60 | |
| 21 | 58.33 | | 56.69 | 56.20 | 55.11 | 54.55 | 53.31 | 52.64 | 52.95 | 54.53 | 57.96 | 59.02 | | 55.57 | | 44892.39 | |
| 22 | 56.40 | | 56.10 | 56.18 | 56.85 | 57.44 | 59.84 | 60.99 | 61.56 | 60.68 | 59.41 | 58.95 | | 58.58 | | 45718.07 | |
| 23 | 47.63 | | 47.66 | 47.24 | 46.02 | 45.30 | 44.91 | 45.58 | 50.20 | 52.99 | 61.48 | 64.43 | | 50.31 | | 47754.25 | |
| 24 | 64.90 | | 65.94 | 66.21 | 66.88 | 67.24 | 68.08 | 68.24 | 68.40 | 67.98 | 67.37 | 67.26 | | 67.14 | | 51102.86 | |
| 25 | 71.39 | | 71.56 | 71.62 | 71.89 | 72.16 | 72.47 | 72.23 | 71.58 | 71.28 | 69.81 | 68.99 | | 71.36 | | 53032.82 | |
| 26 | 38.04 | | 37.53 | 37.56 | 37.79 | 38.04 | 38.64 | 38.90 | 39.19 | 38.56 | 36.98 | 36.84 | | 38.00 | | 54478.18 | |
| 27 | 65.36 | | 65.97 | 66.25 | 67.28 | 67.89 | 69.57 | 70.43 | 73.16 | 74.31 | 76.04 | 75.82 | | 70.19 | | 57332.64 | |
| 28 | 73.57 | | 73.53 | 73.56 | 73.80 | 73.88 | 73.68 | 73.34 | 71.53 | 70.29 | 67.70 | 67.43 | | 72.03 | | 58865.17 | |
| 29 | 63.67 | | 62.62 | 62.49 | 62.04 | 61.83 | 61.35 | 61.31 | 60.62 | 59.59 | 59.57 | 59.91 | | 61.36 | | 65797.75 | |
| 30 | 60.02 | | 59.38 | 59.26 | 58.69 | 58.22 | 56.30 | 55.77 | 55.10 | 54.70 | 54.26 | 54.53 | | 56.93 | | 111408.46 | |
| 31 | 58.04 | | 58.15 | 58.18 | 58.19 | 58.24 | 58.32 | 57.90 | 56.70 | 55.67 | 53.69 | 53.14 | | 56.93 | | 121562.91 | |
| 32 | 64.00 | | 64.11 | 64.08 | 63.79 | 63.49 | 62.59 | 62.31 | 61.52 | 61.17 | 61.14 | 61.15 | | 62.67 | | 147811.64 | |
| 33 | 60.17 | | 60.21 | 60.20 | 60.08 | 59.87 | 59.07 | 58.39 | 56.64 | 55.94 | 54.65 | 54.38 | | 58.14 | | 292057.04 | |
| 34 | 56.15 | | 55.64 | 55.53 | 55.17 | 55.10 | 54.54 | 54.00 | 53.02 | 52.58 | 52.28 | 52.10 | | 54.19 | | 431359.20 | |
| **Mean of each** $\boldsymbol{\theta}$ | 61.51 | | 61.78 | 61.84 | 61.98 | 62.06 | 62.42 | 62.59 | 62.86 | 62.81 | 62.43 | 62.20 | | **PCC** | | **-0.21** | |
